# The Change at Raybrook

**Published:** 1905-08

**Authors:** 


### The Change at Raybrook.

DR. JOHN H. PRYOR, Superintendent of the New York Hospital for Incipient Tuberculosis at Raybrook, in the Adirondack region, has resigned his office. Announcement of this fact was made in the newspapers of July 7, 1905, much to the astonishment as well as regret of all friends of the hospital.

It is a recognised fact that the establishment of this hospital was due largely to the efforts of Dr. Pryor himself, and it is probable that his labors in this direction were rendered without the remotest thought that he would ever become superintendent of the hospital. When it became necessary to fill the place, however, it was apparent to the management that the fittest man to take the initiative in putting the hospital into operation was one of their own number—namely, Dr. Pryor himself, who was therefore appointed.

It was with mingled feelings of pleasure and regret that the citizens of Buffalo, and especially the medical profession, learned of Dr. Pryor's determination to accept the appointment. He was one of our prominent physicians whom we disliked to lose, but we recognised the fact that the opportunity and the man had met.

Dr. Pryor conducted the hospital for a year, and it must be admitted on all hands that it is difficult to see how his work could be improved. In spite of many difficulties and sometimes in the face of bitter opposition at Albany he has achieved a measure of success that is surprising. But Dr. Pryor would not brook delay or interference when the interests of the unfortunates committed to his care were in question. He wanted material for their comfort and cure and he wanted it NOW. And when the fiscal supervisor at Albany denied his reasonable requests, Dr. Pryor told the governor his usefulness was at an end and asked that his successor be appointed.

It is a pity that the state should lose the valuable services of so capable, indefatigable, and honest a public officer as Dr. Pryor, especially for reasons which throw discredit upon methods at Albany for which the state is responsible.

